# Imitation Combined with a Characteristic Stimulus Duration Results in Robust Collective Decision-Making

**DOI:** 10.1371/journal.pone.0140188

**Published:** 2015-10-14

**Authors:** Sylvain Toulet, Jacques Gautrais, Richard Bon, Fernando Peruani

**Affiliations:** 1 Centre de Recherches sur la Cognition Animale, Université de Toulouse, Université Paul Sabatier, Toulouse, France; 2 Centre de Recherches sur la Cognition Animale, Centre National de la Recherche Scientifique, Toulouse, France; 3 Laboratoire J. A. Dieudonné, Centre National de la Recherche Scientifique, Université de Nice Sophia Antipolis, Nice, France; CNRS (National Center for Scientific Research), FRANCE

## Abstract

For group-living animals, reaching consensus to stay cohesive is crucial for their fitness, particularly when collective motion starts and stops. Understanding the decision-making at individual and collective levels upon sudden disturbances is central in the study of collective animal behavior, and concerns the broader question of how information is distributed and evaluated in groups. Despite the relevance of the problem, well-controlled experimental studies that quantify the collective response of groups facing disruptive events are lacking. Here we study the behavior of small-sized groups of uninformed individuals subject to the departure and stop of a trained conspecific. We find that the groups reach an effective consensus: either all uninformed individuals follow the trained one (and collective motion occurs) or none does. Combining experiments and a simple mathematical model we show that the observed phenomena results from the interplay between simple mimetic rules and the characteristic duration of the stimulus, here, the time during which the trained individual is moving away. The proposed mechanism strongly depends on group size, as observed in the experiments, and even if group splitting can occur, the most likely outcome is always a coherent collective group response (consensus). The prevalence of a consensus is expected even if the groups of naives face conflicting information, e.g. if groups contain two subgroups of trained individuals, one trained to stay and one trained to leave. Our results indicate that collective decision-making and consensus in (small) animal groups are likely to be self-organized phenomena that do not involve concertation or even communication among the group members.

## Introduction

Many gregarious vertebrates are fusion-fission species, with frequent changes in size and composition of groups. In addition, the habitat they live in is generally heterogeneous, such that individuals alone or in groups may have to move among more or less distant areas in order to fulfil their basic vital requirements, e.g. drinking, resting or avoiding predators [1–5]. Even during feeding periods, animals have to search for available food, moving slowly and on short distances between feeding stations or more rapidly and on larger distances to exploit distinct feeding areas [6]. Thus, groups either resting or feeding with animals motionless or moving slowly are frequently joined by incoming individuals but also submitted to departures of group members [7–9]. When faced to multiple choices, social organisms must reach consensus in order to maintain the cohesion of the group and the advantages linked to it [3, 10, 11]. The departure of one or few individuals from static groups as well as stops in moving groups compromise social cohesion [12, 13]. This is particularly critical for small groups—a scenario that applies to most gregarious animals [14] despite the popularity and fascination that produce giant bird flocks or fish schools [15–18]—where group splitting represents a serious predatory risk [1].

What influences the individual decisions, *i.e* the interplay between external stimuli and internal state, and which decision-making processes occur to maintain social cohesion are among the most compelling questions in the study of collective animal behavior [5, 19–27]. This goes beyond biology and concerns the broader question of how information at the individual level is evaluated, processed and distributed in the group [28–32]. Certainly, this is highly dependent on the biological system and context we look at. For large groups of gregarious animals on the move such as bird flocks, changes in the moving direction quickly spread through the group in the form of a propagating wave [16–18, 33]. Strong spatial correlations are observed, with local interactions dominating the flock information spreading dynamics [16–18]. Spatial effects are less evident in the behavioral transitions of initially static groups of small sizes, where behavioral shifts are only loosely correlated in space. Recent experiments with primates and fish revealed that an individual spontaneously departing from a static group is likely to give up and return to the group when not followed [7, 24]. It has been also observed that collective motion is promoted by already moving conspecifics [25, 34]. Finally, in some species, a quorum is required to observe collective movement [25, 34–37]. On the other hand, how collective motion stops, remains largely unexplored except for few exceptions [22, 38, 39]. In summary, there is a lack of experimental and theoretical studies focusing on the decision-making processes that trigger and stop collective motion [1, 2, 4, 40].

Here, we use experimentally acquired data to address how groups of uninformed individuals respond to the departure and stop of an informed conspecific in groups of small to medium sizes. We show that the groups of uninformed individuals always reach a consensus: either all uninformed individuals follow the informed one or none does it. Combining experiments where we control the stimulus, associated to the motion of the informed individual, and a mathematical model we unveil that the apparent collective decision-making process leading to an effective consensus results from a self-organized phenomenon resulting from the interplay of simple mimetic rules and the characteristic duration of the stimulus, with group size playing a central role.

## Materials and Methods

### Study area and Experiments

Fieldwork was carried out in the Domaine du Merle (5.74° E, 48.50° N) in the south of France. The field station is located in the Crau region, a very flat area covered by a native steppe. The experiments were performed within irrigated pastures providing homogeneous food resources. From the available flock of 1400 ewes, 30 of them were randomly selected and allocated to the training set and a further 200 ewes to the naive set. All ewes used were unrelated and were 3 to 5 years-old. A number was painted on the back and fleece of each sheep for identification. Both sets were penned up in the same sheepfold during the evening and the night. All the experiments were carried out in daylight (from 10am to 6pm) and the ewes were fed hay in the sheepfold in the morning and in the evening.

### Sheep training

The experiments rely on our capacity to trigger the movement of one sheep towards a fixed panel at the periphery of an arena at a desired time. The protocol used to trained sheep was similar to the one used in previous experiments [41, 42]. Sheep to be trained were originally allocated at random in 6 groups of 5 animals which composition remained unchanged during the training period. Sheep were first habituated in the sheepfold to feed on corn and to receive simultaneously a vibration provided by a neck collar during 3 days. Then the training groups were introduced successively in one of two test arenas (50 x 50m), for a period of 30 to 40 min, each animal wearing a vibrating collar. Ten minutes past the introduction, the collar was activated and one yellow panel (0.5 x 0.5m) was simultaneously raised delivering a handful of corn. Each group received four to eight stimulations, each separated by a period of at least 5 min during each training session. Past 14 days of training, we selected the 3 sheep with the best learning scores (100% of departure toward the panel following a vibration). These 3 trained sheep were comparable in terms of initiation behavior and did not show any differences in movement speed to the panel (ANOVA: $F_{45}^{2}=0.378,P=0.68$). Meanwhile, the set of naive sheep to be used in experiments was confronted to panel rising (without corn delivery) at the periphery of experimental arenas, at one-min interval during two sessions of 90 min. At the end of this habituation session, no naive sheep raised its head when raising the panel. In addition, these two days allowed naive sheep to be familiarized with the experimental setup.

### Experimental procedure

The experimental setup consisted in two arenas (50 x 50m) delimited with sheep fences and surrounded by a visual barrier (propylene net). A 7m-high tower was placed at an equal distance (10m) apart from two next arenas’ corners. Yellow panels (0.5 x 0.5 m) were hammered in the middle of each side for both arenas and were not visible to sheep (S1 Fig). The tests consisted in introducing groups of 8, 16 or 32 sheep within one arena, among which one trained sheep equipped with a vibrating collar. The trained ewes were used no more than twice a day, but were implicated in all group sizes. The naive sheep that composed the rest of the groups were selected randomly for each test. Because of the large number of individuals needed to complete all replications, the naive sheep were used several times, except in groups of 8. A test was conducted as follows: the group was introduced in the arena and sheep grazed spontaneously during 20 min. Then, one of the two panels closest to the tower was raised, waiting for all sheep to graze (*i.e* head down). Simultaneously the vibrating collar of the trained sheep was activated for 2 sec. Past 10 min (end of test), a new panel was raised (one of the two farthest from the tower) to reinforce the conditioning of trained ewes and avoid restricting their space use to the vicinity of the panels closest to the tower. The group was led back to the sheepfold shortly afterward. The naive sheep that were not tested during one experimental day were introduced in distant pasture. We never performed two trials in parallel. We also carried out control experiments to be sure that naive ewes did not associate the panel rise and the food reward. Thereby, 6 tests before and 6 after the test series were conducted with groups of 32 naive ewes, using the same protocol as described before. We found no movement of groups when raising the panel, almost all sheep continuing their spontaneous activity.

### Data collection and analyses

Two digital cameras (Canon EOS D50) were fixed on the tower, each one focusing on one arena. Fifteen minutes after the introduction of the groups, the digital camera was turned on, taking a picture of the entire arena every second and turned off five minutes after the panel was raised. For each replication, we obtained a series of about 600 pictures. Using a custom software developed by JG [43, 44], we were able to track on each picture the position and the orientation of animals by dragging a vector on their back, and identify the behavior of each individual, *i.e* grazing, standing head-up, moving and others.

We defined a departure of the trained sheep (initiator), *i.e* initiation past experimental stimulation when it performed an uninterrupted walk towards the raised panel. The following behavior, *i.e* a new departure, was defined as the movement of a naive ewe, occurring after the trained sheep departure, without stop until joining the trained individual near the panel. The behavior of stopping was defined as an individual ceasing to walk and remaining either stationary head-up or grazing. Six replications in the groups of 32 were discarded, one because the initiator did not depart, two because the initiator stopped moving between the group and the target and three because the initiator showed a moving behavior not comparable to other trials (going to a wrong target first and then joining the rewarded target). Thus we performed analyses on 15 trials for groups of 8 and 16 sheep and 24 trials for groups of 32.

The level *α* was fixed to 0.05 for statistical significance. All analyses were conducted using R version 3.0.1.

### Ethics statement

All the animals were maintained under routine husbandry conditions at a Montpellier Supagro research station (Domaine du merle, Salon-de-Provence, France) with full approval of its director Pierre-Marie Bouquet. Animal welfare requirements were fully respected in accordance with the European Directive 2010/63/EU, with the rules of the European Convention for the Protection of Vertebrate Animals used for Experimental and Other Scientific Purposes and with the Convention of the French Comité national de réflexion éthique sur l’expérimentation animale. No special authorization from the French Ethical Committee for animal experimentation (Commission nationale de l’expérimentation animale) was required as no protected or endangered species was involved, as the experiments did not imply any invasive manipulation (the experimental protocol consists in the observation of groups and the acquired data are only pictures of the animals) and as sheep were conducted to the test arenas, as they are herded on a daily basis to the pastures. All personnel involved had technical support from the employees of the research station as required by the French Ministry of Research. The experimental protocols included short test periods (20 minutes) where sheep did not experience painful, stressful or unfamiliar situations. The experimental procedures had no detrimental effect on the sheep and at the end of the experiment all the animals reintegrated the sheep herd of the breeding research station.

## Results and Discussion

**Fig 1:**
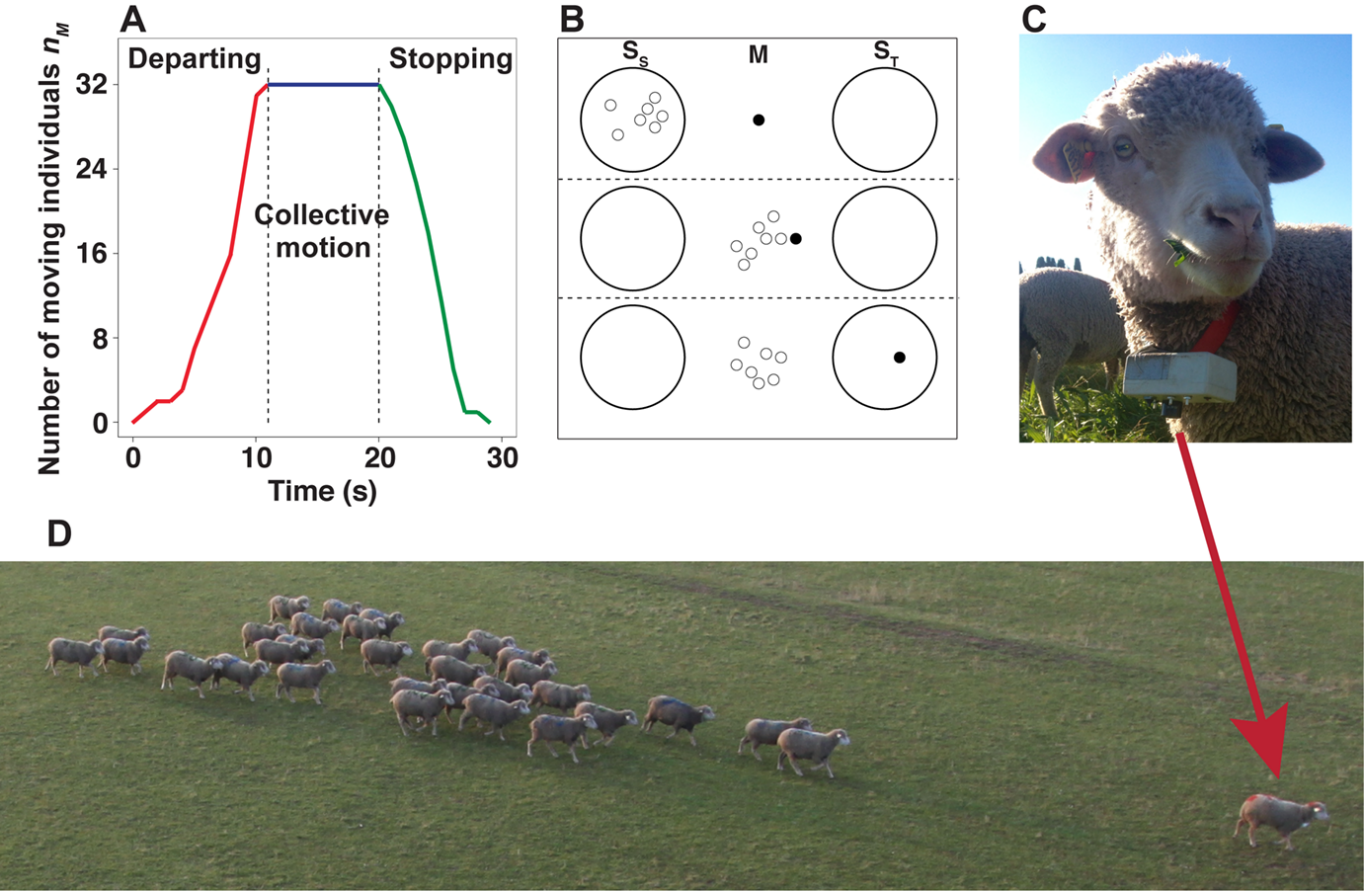
Experimental collective observations. (A) The number of moving individuals (n_M_) as a function of time in one of the trials with 32 sheep. The departing (in red), collective motion (in blue) and stopping (in green) phases are indicated. (B) Sketch illustrating the temporal phases of an experiment. The three behavioral states of individuals are represented: (static) individuals at the starting position (S_S_), moving (M), and stopped near the target (S_T_). The initiator is depicted by a full circle, while open circles correspond to naive individuals. From top to bottom, we observe the first transition S_S_ → M, the collective motion phase, and the first transition M → S_T_. (C) shows one of the trained individual fitted with the vibrating collar. (D) A snapshot of a herd of 32 sheep in collective motion provoked by the departure of the trained individual.

In our experiments, we work with groups of N = 8, 16 and 32 sheep, among which 1 is a trained individual—henceforth referred to as initiator—while the remaining N—1 are uninformed/naive individuals. The initiator is trained to move towards a target located at the periphery of the arena when a vibrating collar is activated by a remote control (Fig 1C). The group is subject to the perturbation produced by the initiator: *i.e.* the sudden departure and stop of the initiator, which challenge the social cohesion of the group. When the departure of the initiator triggers a collective response, we observe three distinct consecutive phases: departing, collective motion, and stopping as illustrated in Fig 1A. The departing phase starts with the departure of the initiator and continues until the number n_M_ of moving individuals increases to match the group size. At this point, the collective motion phase begins, with the group moving cohesively behind the initiator. The stop of the initiator near the target marks the onset of the stopping phase, where n_M_ decreases until reaching 0. The behavior of an individual can be characterized by one of the following states: stopped at the starting position (S_S_), moving (M), or stopped at the target position (S_T_) (Fig 1B). The whole process can be then described as a transition first from S_S_ to M, and then from M to S_T_.

In all trials with 8 and 16 sheep, the departure of the initiator systematically triggers a collective motion (Fig 1D). In groups of 32 the departure of the initiator does not always lead to a collective motion of the naive group. Splitting of the naive group has not been observed (see S1 and S2 Movies for examples.)

**Fig 2:**
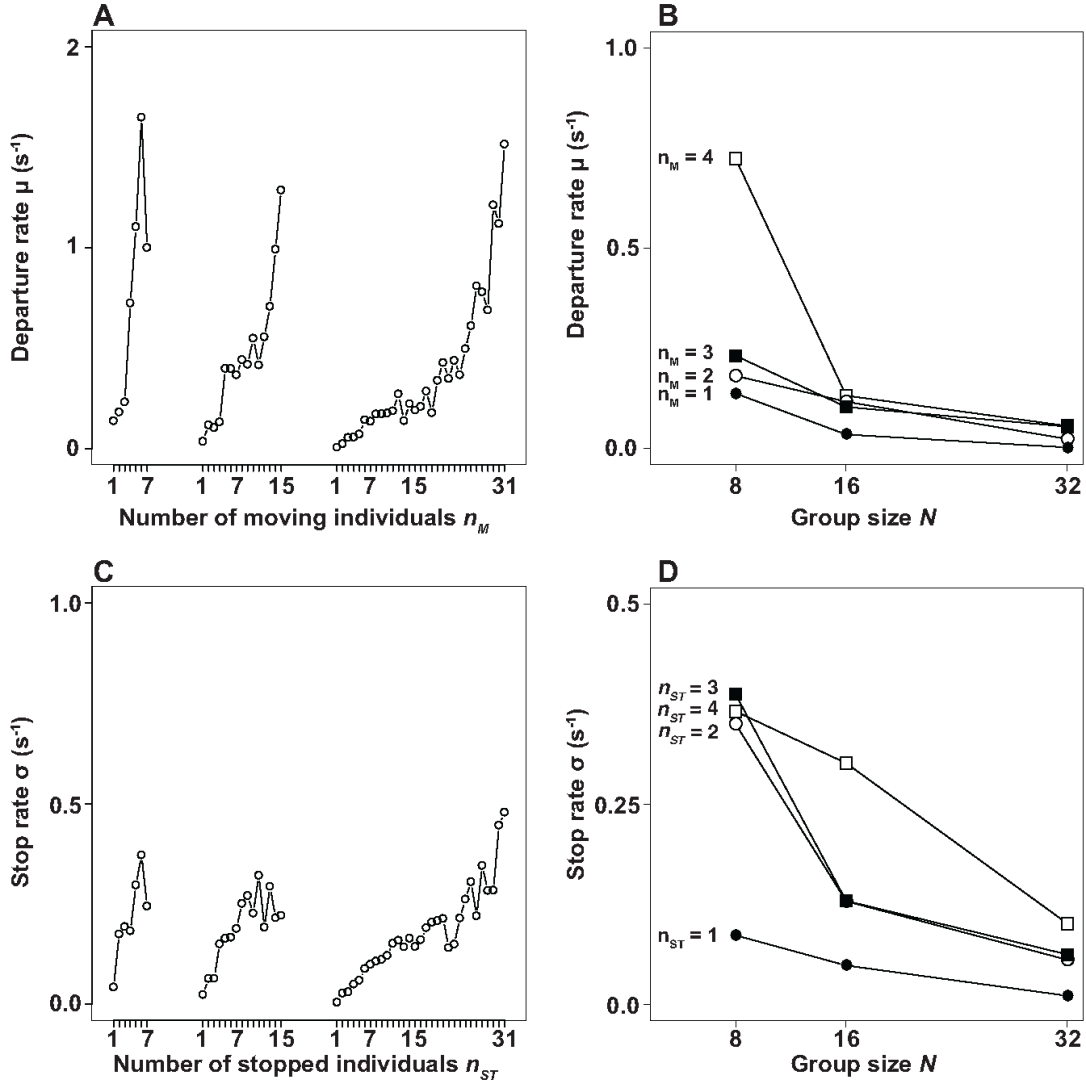
Individual transition rates. (A) and (C) show that the departure and stop rate increase with n_M_ and n_S_T__, respectively, for all group sizes (N = 8, 16 and 32). (B) The inhibiting effect of S_S_ on the transition S_S_ → M for a given n_M_ is evidenced by the decrease of the transition rate with N. (D) Similarly, in the transition M → S_T_, we observe a decrease of the transition rate with N for a fixed value of n_S_T__, which indicates an inhibiting role of n_M_.

We start our analysis by focusing first on the cases where the initiator successfully provokes a collective motion. We are interested in quantifying the decision of sheep to switch from S_S_ to M and from M to S_T_. To account for the dynamics of the departing and stopping phases, we focus on the individual transition rates (the probability per time unit for a given individual to switch behavior). From the experimental data we estimate the departure (*μ*) and stopping (*σ*) rates for each departure and stopping rank, respectively (see S3 Text for details on the computation). Fig 2A (respectively, Fig 2C) shows that the individual transition rate *μ* from S_S_ to M (in Fig 2C, *σ* from M to S_T_) increases with n_M_ (with n_S_T__, the number of individuals in state S_T_, in Fig 2C). Fig 2B (respectively, Fig 2D) indicates that the transition rate *μ* from S_S_ to M (respectively, *σ* from M to S_T_) for a fixed value of n_M_ (fixed value n_S_T__, for M → S_T_) diminishes with *n*_*S*_*S*__ ∼ *N* − *n*_*M*_ (*n*_*M*_ ∼ *N* − *n*_*S*_*T*__ in Fig 2D). This indicates that both transitions S_S_ → M and M → S_T_ share similar features: they both exhibit a promoting component (n_M_ in S_S_ → M and n_S_T__ in M → S_T_) and an inhibiting component (n_S_S__ in S_S_ → M and n_M_ in M → S_T_). These findings are consistent with previous studies on the transition to departure using smaller group sizes [41, 42]. Notice that we have presented Fig 2B and 2D in such a way that the dependency of the individual transition rates *μ* and *σ* with the group size *N* becomes explicit and evident. This observation is a clear indication that the individual transition rates are not strongly limited by a local neighborhood of interaction. The presence of (very short range) local interaction leading to an average number of interacting neighbors (per individual) significantly smaller than the group size N, would lead to a quick saturation of *μ* and *σ* with N (*i.e.* to a “reservoir” limitation), and the suppression of the observed group size effect. In practice, this indirectly means that we are likely to be close to a situation where every individual is able to perceive most members in the group, *i.e.* we are in the vicinity of global perception. This does not mean that individuals exhibit infinite perceptual and cognitive capacities, but simply that the spatial extension of the group as well as the group size are within the perceptual and cognitive limitations of the animals. This should not be taken as a serious limitation of the current study, given the fact that many gregarious animals live in small-size groups [14] and are likely to remain always in the vicinity of global perception most of the time.

**Fig 3:**
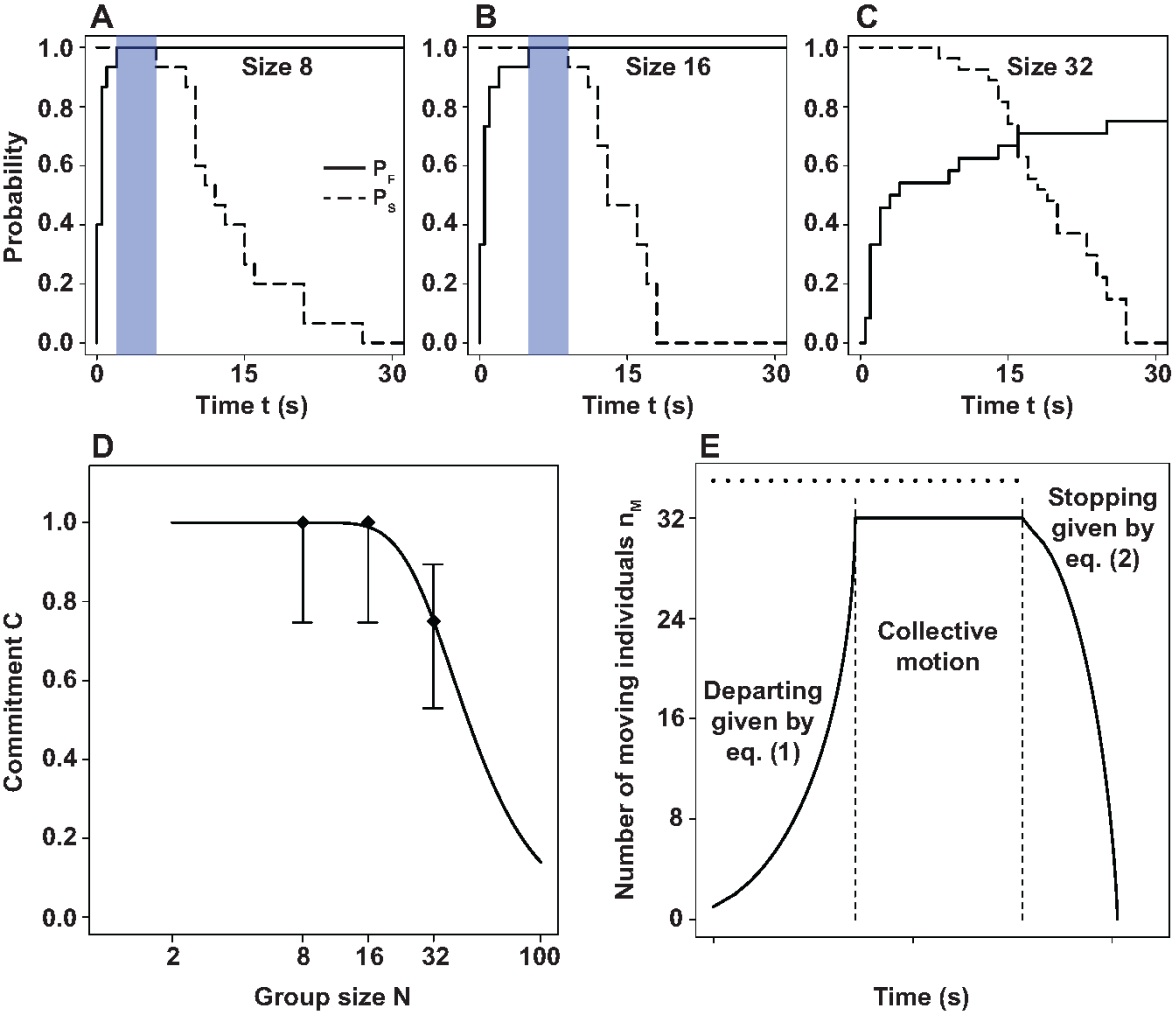
Following latency versus stimulus duration and model fitting. (A-C) Probability P_F_ of observing a first follow event before time t (solid line) and probability P_S_ that the initiator has not reached the target at t (dashed line) for group sizes N = 8, 16, and 32. The blue areas indicate the time window between the maximum departure time for the first follower and the minimum required time for the initiator to reach the target. (D) Probability that the initiator is followed by at least one naive sheep (referred to as commitment) as a function of group size N in experiments (black diamonds) and as predicted by Eq (3) (black line). Whiskers represent 95% confidence intervals. (E) Predicted values of n_M_(t)) for N = 32. The dotted line indicates the time required by the initiator to reach the target (*τ*).

As mentioned above, the departure of the initiator does not always trigger collective motion in groups of 32 individuals. Importantly, the absence of collective follow is not related to a peculiar behavior of the initiator. In particular, all initiators displayed highly direct trajectories towards the target (in contrast to what has been found in trained fish in [33]) and we found no impact of their velocity on the collective outcome (see S1 Text for details). These observations suggest a social effect linked to group size (*i.e* to the number of uninformed individuals). For groups with N = 32, the uninformed individuals, upon departure of the initiator, respond in an all-or-none way: either all follow the initiator or none of them does, and thus no fission of the naive group is observed. This phenomenon, which seems at first glance to require some sort of concertation among the naive individuals, can be understood by focusing on the behavior of the first potential follower. Our argument is based on the assumption that the initiator can stimulate a transition S_S_ → M only when moving to the target position, *i.e.* while being in state M. This implies that if no naive sheep departs by the time the initiator reaches the target, no transition S_S_ → M will ever occur. In consequence, the probability P_S_ that the initiator is still moving at time t provides a rough estimate of the probability that the stimulation is still present. All this means that the problem can be reduced to the competition between two probabilities: P_S_ and the probability P_F_ that the first follower departs before time t, as illustrated in Fig 3A–3C. Fig 3A and 3B indicate that for N = 8 and 16, the transition S_S_ → M for the first followers always occurs before the initiator stops near the target position. On the other hand, Fig 3C shows that the transition S_S_ → M for the first follower is such that the initiator can stop at the target position before this transition has ever occurred. This provides a qualitative explanation of the remarkable group size effects observed in the experiments.

Now, we go further in the quantitative analysis using a mathematical model, which has proved useful to analyze the experimental data and to test the various hypotheses formulated to interpret the observed phenomena. Given the strong group size effects exhibited by the individual transition rates *μ* and *σ* (Fig 2), we build the model assuming that each individual is able to perceive all individuals in the group. Such global perception assumption has to necessarily break down above a given group size N_*_, and model predictions are only reliable for *N* < N_*_, becoming less accurate as group size approaches N_*_. While we ignore the actual value of N_*_ for our experiments, the obtained results strongly suggest that *N* < N_*_ (meaning N_*_ > 32). Under these assumptions, we will see that (i) the departure and stopping rates can be expressed as non-linear functions, with promoting and inhibiting components as proposed above, and (ii) that the hypothesis that the initiator can only induce a transition S_S_ → M while being in state M is consistent with the experimental data. Our first step is to formulate the (individual) rate *μ* (which is a probability per time unit per individual) associated to the transition S_S_ → M as:

$$\begin{array}{c} \mu \left(n_{M},n_{S_{S}}\right)=\alpha \frac{n_{M}^{\beta }}{{n_{S_{S}}}^{\gamma }} \end{array}$$ (1)

with *α*, *β* and *γ* parameters modulating the effect of n_S_S__ and n_M_ on *μ*. Given expression Eq (1), we can compute the mean time to depart t(n_M_) for the n_M_^th^ follower, assuming that *n*_*S*_*S*__(*t*)+*n*_*M*_(*t*) = *N*, as $t(n_{M})=\sum_{n=1}^{n_{M}}\tilde{\mu }{\left(n,N\right)}^{-1}$, where $\tilde{\mu }=\mu (n,N)\;(N-n)$ is the departure transition rate at the group level. From the inverse function of this expression we obtain n_M_ as a function of time (Fig 3E), *i.e.* from t(n_M_) we obtain n_M_(t). Notice that the departing phase is then given by $\sum_{n=1}^{N-1}\tilde{\mu }{\left(n,N\right)}^{-1}$, which means that the average collective motion phase is approximately $\tau -\sum_{n=1}^{N-1}\tilde{\mu }{\left(n,N\right)}^{-1}$, where *τ* is the time required by the initiator to reach the target (see S5 Text).

In analogy to Eq (1), we assume that the (individual) stop rate *σ*, related to the transition from M → S_T_, is given by

$$\begin{array}{c} \sigma \left(n_{M},N\right)=\alpha^{\prime }\frac{{n_{S_{T}}}^{\beta^{\prime }}}{{n_{M}}^{\gamma^{\prime }}}=\alpha^{\prime }\frac{{\left(N-n_{M}\right)}^{\beta^{\prime }}}{{n_{M}}^{\gamma^{\prime }}} \end{array}$$ (2)

with *α*′, *β*′ and *γ*′ parameters modulating the effect of n_M_ and n_S_T__ on *σ*. During the stopping phase, n_M_(t) is obtained, under the assumption that *n*_*M*_(*t*)+*n*_*S*_*T*__(*t*) = *N*, from the inverse function of $t(n_{M})=\tau +\sum_{n=N-1}^{n_{M}}\tilde{\sigma }{\left(n,N\right)}^{-1}$ with $\tilde{\sigma }=\sigma (n,N)\;n$, where *n* ≤ *N* − 1.

We emphasize that in the model we propose, the transition rate to switch behavior depends only on the number of individuals in the two involved behavioral states. This means for example that the probability to switch to M for an individual in S_S_, does not depend on the number of individuals in S_T_.

As commented above, it can occur that uninformed individuals do not follow the initiator in groups of N = 32. Using the proposed mathematical framework, we can account for such group size effect. We recall that the absence of collective motion is associated with those situations where the initiator does not induce any transition S_S_ → M of a naive individual during the time *τ* required by the initiator to reach the target position. Mathematically, the problem reduces to compute the probability C—henceforth referred to as *commitment*—to observe a transition S_S_ → M of a naive individual during *τ*, which takes the form:

$$\begin{array}{c} C=1-\frac{1}{\left(\tau_{max}-\tau_{min}\right){\tilde{\mu }}_{i}}\left(e^{-{\tilde{\mu }}_{i}.\tau_{min}}-e^{-{\tilde{\mu }}_{i}.\tau_{max}}\right) \end{array}$$ (3)

with ${\tilde{\mu }}_{i}=\alpha \;i^{\beta }{\left(N-i\right)}^{1-\gamma }$, i the number of initiators (i = 1 in our experiments) and *τ*_*min*_ and *τ*_*max*_, the minimum and maximum observed *τ* values, respectively (see S6 Text for the derivation of expression Eq (3)). The estimation of parameters is done in two steps. The best fit of Eq (3) to the experimental points in Fig 3D provides a set of *α* and *γ* values. Fixing these two parameters, the best fit on the individual rates is used to estimate the other parameter. More details on the fitting procedure are given in S4 Text. The obtained values are *α* = 90.1, *β* = 2.5, *γ* = 3, and *α*′ = 0.23, *β*′ = 0.53, *γ*′ = 0.41. Fig 3E shows that the dynamics resulting from Eqs (1) and (2) provides a qualitative description of the data (*cf.* Fig 1A), while Fig 3D shows that the model also accounts for the experimentally observed commitment C. It worth noticing that given the nonlinear form of the transition rates and given the obtained parameter values, it turns out that after a first transition S_S_ → M of a naive individual, a cascade of transitions S_S_ → M will immediately follow.

**Fig 4:**
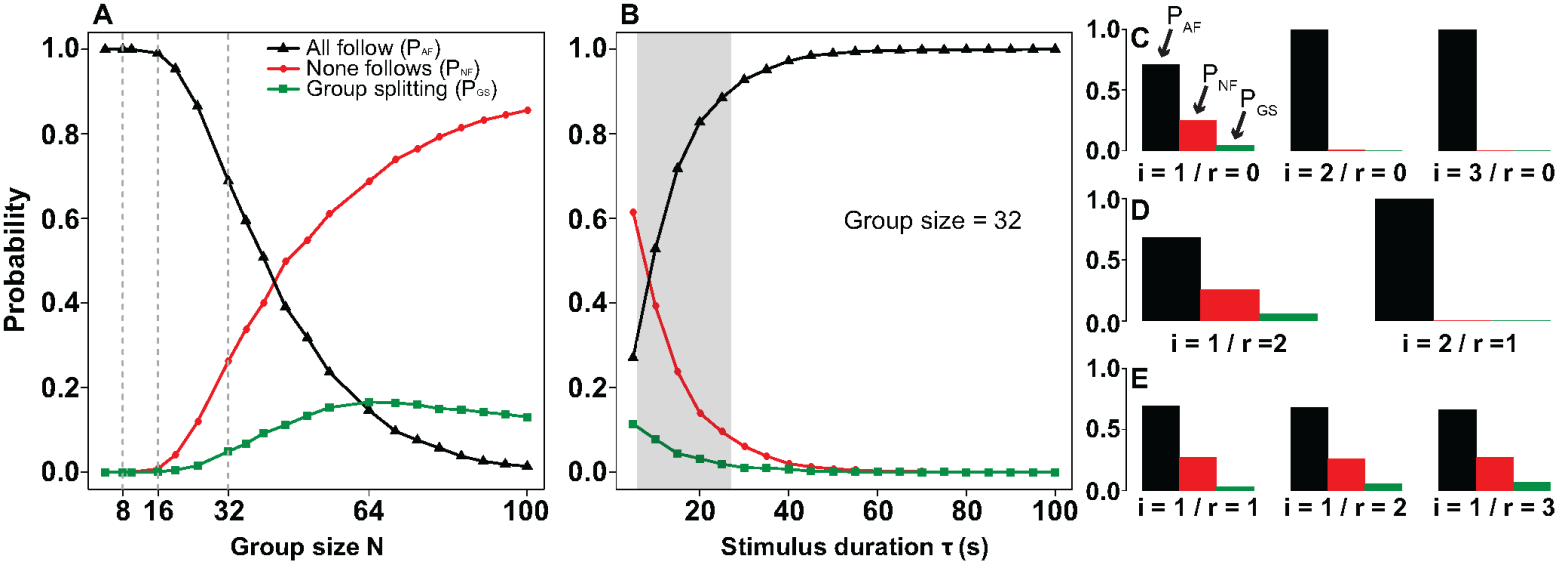
Model predictions for collective decision of naives. (A) Probabilities P_AF_ of observing a full collective follow (black line), P_NF_ corresponding to no follow (red line), and P_GS_ to group splitting (green line) as a function of group size N for groups of naives subject to the departure of 1 initiator. (B) P_AF_, P_NF_, and P_GS_ as function of the stimulus time, associated to the time required by the initiator to reach the target position, for N = 32 including 1 initiator. The grey area shows the interval of stimulus durations we observed experimentally. (C), (D) and (E) show P_AF_, P_NF_, and P_GS_ when the naive group is faced to contradictory information cues: a number i of individuals determined to go towards the target (initiators) and the number r of individual determined to stay (see text for details). Results for N = 32. Notice that i and r do not induce a symmetric collective bias.

The mathematical model allows exploring a large variety of hypothetical scenarios, using conditions going beyond the ones used experimentally. It is important to stress that model predictions are reliable as long as all hypotheses and assumptions remain valid. This implies, among other things, that results are reliable exclusively for group sizes *N* such that *N* < N_*_, with the critical group size N_*_ > 32. Ultimately, models predictions should be verified/falsified by performing further experiments. Always according to our mathematical model, there exist three possible outcomes when the naive group faces the departure of one or more initiators: i) all naive individuals follow the initiator(s), ii) none of them does, and iii) only a fraction of naive individuals follow the initiator(s). The associated probabilities, P_AF_ for i), P_NF_ for ii), and P_GS_ for iii), are shown in Fig 4. While the first two scenarii, *i.e.* i) and ii), imply a sort of consensus for the naive group, which assures group cohesion, the third scenario, *i.e.* iii), implies group fission. Although according to the mathematical model, group splitting can occur, the associated probability is always small. Fig 4A shows that in groups of *N* ≤ 32, the probability of observing group splitting is smaller than 5%. Given that with N = 32 we have performed 24 experimental trials, one could expect in mean one group splitting event. In short, the experimental observations are consistent with model predictions. In addition, the model predicts—for a fixed range of *τ* (*i.e.* stimulation duration)—that full collective motion decreases non linearly with group size N, while the probability P_NF_ that none follows increases non linearly (black and red curves, respectively, in Fig 4A). The probability of observing splitting of the naive group (green curve) exhibits a non monotonic dependency with N with a maximum at N = 64, where the probability is close to 20%. This means that by performing similar experiments with 63 naive individuals and one initiator—and performing a similar number of replications of the experiment—we should be able to observe group fission. It is worth noticing that if the group size is larger than 64, the probability of splitting decreases again. Furthermore, the model predicts that for large group sizes the departure of the initiator cannot induce naive individuals to move towards the target position: for large values of *N* the naive group remains unresponsive to the departure of the initiator. This observation is, however, only valid for a fixed range of *τ*. If we imagine that the experiment is performed in larger arenas, in such a way that the target position is located farther away and *τ* is significantly larger, the departure of the initiator will again lead naive individuals to move towards the target (Fig 4B). This is in line with recent results [16, 33] that suggest that an increase in the stimulus duration enhances the collective response. The model also predicts that if the *τ* is large enough, the only possible outcome upon the departure of the initiator is a full collective follow. Accordingly, the only scenario where fission of the naive group can be observed is when the two resulting subgroups will be separated a relatively small distance one from the other, at which point we could ask ourselves whether such separation qualifies truly as group splitting.

Finally, the model also allows to explore situations where naive groups are subject to conflicting information cues. For instance, let us imagine we trained a group of i individuals to move towards the target position and a group of r individuals to stay at the starting position. We are interested in the behavior of the naive individuals, for whom there are always two mutually exclusive options—either to stay or to go –, which means that all probabilities, P_AF_, P_NF_, and P_GS_, are computed with respect to the naive group. To avoid confusions, we clarify that this implies that P_AF_ is the probability that all naive individuals follow, P_NF_ none of the naive individuals follows, and P_GS_ the probability that at the end of the process we find some of the naive individuals at the starting position and some at the target position. We start out with a simple scenario where there is no trained individual to stay, *i.e.* r = 0. By increasing the number i of individuals moving away, we find, not surprisingly, that P_AF_ increases dramatically, to the point that the only possible outcome is a collective motion (*i.e.* P_AF_ → 1, Fig 4C). Similarly, we can fix i and increase the number r of individuals determined to stay. We find that increasing r leads to a very weak increase of P_NF_ and P_GS_ (Fig 4E). Thus, there is a clear asymmetry in the role played by i and r. This is particularly evident by fixing the number of trained individuals and varying the relative weight between i and r. Fig 4D corresponds to i + r = 3 and shows that i = 1 and r = 2 is remarkably different from i = 2 and r = 1. In particular, while for i = 2 and r = 1, P_AF_ → 1, for i = 1 and r = 2, we do not find that P_NF_ → 1. Moreover, i = 1 and r = 2 differs only slightly from i = 1 and r = 0. This means that if we have initially i = 1 and r = 1, adding an extra trained individual determined to move towards the target ensures a full collective follow, while adding an extra individual determined to stay has a very weak effect on the collective outcome. We have to make r close to N to ensure that the trained individual will systematically fail to recruit any naive sheep. In short, a small variation of r has a weaker effect than a small variation of i. Adding individuals determined to either stay or to leave does not produce, at the collective level, a symmetric bias, even if staying and leaving are opposite alternatives, naive individuals can only perceive the stimulation that constitute the moving individuals. The r individuals do not move and thus do not differ from other stopped individuals (the naive ones). The only effect of the r individuals is to slow down the departure dynamic. This is in sharp contrast with binary decision studies [25, 45–47] where mutually exclusive options are considered as symmetric. This is particularly clear in flocking models [46, 47] where left-right choices are such that individuals determined to move towards the left exert the same social influence than individuals determined to move towards the right. One important message we learn is that opposite alternatives are not necessarily symmetric. The origin of such asymmetry may be related to the fact that at the individual level the decision whether to stay or to leave can be formulated as a decision whether to remain in the current state or to change it. The obtained results, at both the experimental and theoretical level, suggest that *behavioral change* is strongly favored. Moreover, it seems that the individuals that initiate a change become—as previously proposed [48]—incidental leaders, while those determined to remain in the current state, though playing an inhibiting role, exert a weaker influence on the naive group.

## Conclusions

Here, we have shown that simple mimetic responses—as those described by Eqs (1) and (2)—when combined with the characteristic duration associated to the stimulus—here, the time required to arrive at the target position—act as an effective collective decision-making mechanism. Moreover, we showed that the proposed mechanism, whose derivation is intimately based on the presented experimental observations, and valid exclusively for small group size where spatial effects can be neglected, allows a group of naive/uninformed individuals to solve a scenario with conflicting information in such a way that the most probable collective outcome is a consensus. Specifically, we analyzed a situation where the naive group faces a scenario where there is a subgroup decided to stay and a subgroup decided to go, and showed that the two most probable outcomes are that either all naive individuals follow or none does. Importantly, though group splitting cannot be discarded, such event is, according to the proposed mechanism, unlikely.

In summary, the interplay between mimetic rules and the characteristic duration of the stimulus leads to a self-organized collective decision-making that does not required neither explicit communication nor concertation among the group members. While this observation is likely to hold even for large groups, the strong group size effects reported here (see Fig 3) suggest that the group is in the vicinity of global perception, a situation that requires the group to be relatively small in size. This is in sharp contrast to what is expected for largely extended groups (with respect to the perception and cognitive limitation of the individuals) or in densely packed groups in motion, where collision avoidance leads necessarily to strong local spatial effects [15–18, 49]. In these two scenarii, large group sizes or dense groups in motion, we conjecture a saturation of the behavioral response of individuals given by a local interaction neighborhood, leading to the absence of the here reported group size effects. In such scenarii, the group dynamics is likely to strongly depend on the spatial structure of the group as suggested in [15–18, 49].

Finally, it is worth noticing that the effective decision-making mechanism described here applies to a specific social context: a group of naïve individuals sharing an initial behavioral state, which is subject to the behavioral shift of one or several conspecifics. One of the essential elements of the proposed mechanism is the presence of a discrete number of behavioral states, which has to be, necessarily, larger than two (as *e.g.* S_S_, M and S_T_). This means that models for groups in motion, where the group has to decide in which direction to move [46] cannot be used to model the specific social context addressed here. Such models have been designed to describe the navigation of a group, and not to describe behavioral shifts. At the mathematical level the differences are evident. While navigation models associate a continuous variable to each individual, related to the moving direction of the individual, behavioral shift models deal with discrete variables associated to the possible behavioral states of the group members. Similarly, the social mechanism analyzed here cannot be directly compared with decision-making models designed to describe dichotomic decisions where the initial state differs from the two (opposite) final ones the individuals are forced to choose [9, 25]. This is in sharp contrast with the social context we are interested in here, where individuals initially share the same behavioral state and the options reduce to either remain in this initial state or to switch to the other available state. In summary, the collective decision-making mechanism proposed here is fundamentally different, and not comparable to previous collective decision-making mechanisms, which have been designed to describe a different social context. Though deeply rooted in the models proposed in [34, 41], the mechanism described here differs from these ones by making use of three behavioral states, accounting simultaneously for the initiation and stop of the collective response, and with both processes modeled as a transition at the individual level.

Given the simplicity of the proposed mechanisms here, we expect similar mechanisms to be at work in other animal systems.

## Supporting Information

S1 Text Details on the behavior of sheep. (PDF)

S2 Text Statistics of the departing and stopping phases. (PDF)

S3 Text Details on the estimation of the experimental rates. (PDF)

S4 Text Details on the parameter estimation. (PDF)

S5 Text Details on the calculation of predicted n_M_(t). (PDF)

S6 Text Details on the calculation of the commitment (Eq (3)). (PDF)

S1 Table List of times obtained by Eq (S5.4). (PDF)

S2 Table List of times obtained by Eq (S5.10). (PDF)

S1 Fig Experimental setup. The setup is composed of two arenas (50 m side) delimited in native irrigated pasture and surrounded by a 1.2 m visual barrier (propylene net). Observations were made possible thanks to an observation tower located nearby. Yellow panels placed in the middle of each side can be levelled up and used as targets for the trained sheep thanks to a remote control. Digital snapshots were taken at one-second interval with a camera fixed at the top of the tower.
(EPS)

S2 Fig Statistics of the departing phase. (A) Latency to depart of the first followers as a function of the group size. (B) Duration of the departing phase i.e. time elapsed between the departure of the first and the last followers. The bottom and top of “boxes” show the first and third quartile of data. The thick lines show the median and the whiskers represent the minimum and maximum values of the distribution.
(EPS)

S3 Fig Statistics of the stopping phase. (A) Latency to stop of the first stoppers as a function of the group size. (B) Duration of the stopping phase i.e. time elapsed between the first and the last stopping events.
(EPS)

S4 Fig Survival curves of the latencies of the first followers in groups of 32 without and with censored data (adding the times spent walking by initiators to reach the target in trials when they fail to be followed) Dashed curves show the experimental data without (black curve) and with (red curve) the censored data. Respectively plain black (*e* ^−0.19*t*^; time constant = 5.26 s) and red lines (*e* ^−0.08*t*^; time constant = 12.56 s) show the exponential curves fitted to the corresponding experimental data. Red crosses: the times spent walking by initiators to reach the target in trials when they fail to be followed. Dotted lines: ± 95% confidence interval.
(EPS)

S5 Fig Model predictions. Probability C of triggering collective motion (commitment) as a function of group size for various numbers i of initiators (A), and as a function of i for a fixed group size (B). (C) The minimum number of initiators required to always observe a follow (commitment = 1) as a function of group size (black line). The grey area indicates the combination of initiators and group size where the commitment is less than 1 (risk zone: none follows the initiator).
(EPS)

S1 Movie One example of a trial in a group of 32 where the initiator triggers a collective motion. We compiled the snapshots at a rate of 2 frames per second (1 second in the video represents 2 s in real time). At 12 s, we added at the bottom left, for each frame a figure showing the locations of the group members across time. The initiator is plotted in blue. Naive individuals not moving are plotted in black and the moving ones in red.
(MOV)

S2 Movie One example of a trial in a group of 32 where the initiator fails to provoke a collective motion. Parameters as in S1 Movie.
(MOV)

S1 File Experimental data for departures. Each row corresponds to an individual in each replication. Columns: “Replic” = Replication number; “Initiator” = Identity of the initiator; “Ident” = Identity of the individual; “Latency” = Departure latency since the initiator departure; “Departure_Rank” = Departure rank; “Status” = Status of the individual (“t” = initiator and “f” = naive individual); “n_M” = Number of individuals in M when the individual departs; “n_SS” = Number of individuals in S_S_ when the individual departs; “Size” = Group Size.
(TXT)

S2 File Experimental data for stops. Each row corresponds to an individual in each replication. Columns: “Replic” = Replication number; “Initiator” = Identity of the initiator; “Ident” = Identity of the individual; “Latency” = Departure latency since the initiator stop; “Stop_Rank” = Stopping rank; “Status” = Status of the individual (“t” = initiator and “f” = naive individual); “n_ST” = Number of individuals in S_T_ when the individual stops; “n_M” = Number of individuals in M when the individual stops; “Size” = Group Size.
(TXT)
