# Supplementary material for: Imitation Combined with a Characteristic Stimulus Duration Results in Robust Collective Decision-Making
Source: PLoS One. 2015 Oct 14;10(10):e0140188. doi: 10.1371/journal.pone.0140188 (PMC4605660; doi:10.1371/journal.pone.0140188)
Supplement: S1 Text — (PDF) [file pone.0140188.s001.pdf]

S1 Text

Details on the behavior of sheep. In trials with groups of 8 and 16, the initiation always triggered the departure of all naives. In groups of 32, only 75% of the trials (18 out of 24) displayed such collective response. We investigated whether the lack of commitment of the naives could be explained by behavioral differences in initiations or by particular group configuration. We used three different initiators in the experiments that were tested in all group sizes. Each failed to entrain naive sheep i.e. the cases where no collective departure was observed cannot be due to any particular trained sheep. When comparing trials with and without follower, no differences were found regarding initiators' movement mean speed (Student t-test: T = -1.08, P = 0.3) or in terms of group density (Wilcoxon test: W = 66, P = 0.9). Thus, the lack of commitment cannot be explained by any peculiar behavior or position of the initiators, indicating that social mechanisms are involved. Trials without collective motion occurred on days 3, 4, 8, 15 and 16 of the 17 days of experiments discarding any potential effect of habituation or change in motivation of the naives. All naive sheep were tested in control trials in groups of 32 (without trained individual), 6 before and 6 after the experiments. These control groups were confronted to a panel rise in the same conditions as in the experimental trials (except that no sheep was wearing a vibrating collar). No naive sheep responded to the panel rise *i.e.* we did not record any movement nor any behavioral modification.
