# Supplementary material for: Imitation Combined with a Characteristic Stimulus Duration Results in Robust Collective Decision-Making
Source: PLoS One. 2015 Oct 14;10(10):e0140188. doi: 10.1371/journal.pone.0140188 (PMC4605660; doi:10.1371/journal.pone.0140188)
Supplement: S2 Text — (PDF) [file pone.0140188.s002.pdf]

S2 Text

Statistics of the departing and stopping phases. We tested whether latencies of the first followers were affected by group size. We found a significant effect of group size (medians: 1 s, 1 s and 2 s for groups of 8, 16 and 32 respectively; Kruskal-Wallis test: $\chi^2 = 8.67$, df = 2, P = 0.01). Furthermore, the first followers' latencies were more variable in groups of 32 than in smaller ones (Bartlett-test: K2 = 42.97, df = 2, P < 0.0001; S2 Fig.A). The duration of the departing phase (time elapsed between the first and the last following events) increases with group size (medians: 3.6 s, 7 s and 14.5 s respectively; $\chi^2 = 24.51$, df = 2, P < 0.0001) but also varied highly in the larger group size ($K^2 = 20.81$, df = 2, P < 0.0001; S2 Fig.B). With the same logic, we tested the effect of group size on the latencies of first stoppers. There is no significant effect of group size on the first stop latency (medians: 1.3 s, 1.3 s and 3 s respectively for group sizes 8, 16 and 32; $\chi^2 = 4$, df = 2, P = 0.13), but stop latencies were more variable in groups of 32 ($K^2 = 10.14$, df = 2, P = 0.006; S3 Fig.A). The duration of the stopping phase (time elapsed between the first and the last stop) increases (medians: 6 s, 7.5 s and 11 s respectively; $\chi^2 = 12.28$, df = 2, P = 0.002) but the variance was not affected by group size ($K^2 = 4.12$, df = 2, P = 0.13; S3 Fig.B).
