# Supplementary material for: Imitation Combined with a Characteristic Stimulus Duration Results in Robust Collective Decision-Making
Source: PLoS One. 2015 Oct 14;10(10):e0140188. doi: 10.1371/journal.pone.0140188 (PMC4605660; doi:10.1371/journal.pone.0140188)
Supplement: S3 Text — (PDF) [file pone.0140188.s003.pdf]

S3 Text

Details on the estimation of the experimental rates. The transition rates in Fig. 2 are estimated from the experimental data. Let us take an example of how the departure rate (probability per unit of time for an individual to depart) is estimated for groups of 32 individuals. For a given departure rank, $n_M = k$ (and then $n_{S_S} = N - k$) we have 18 (the number of experimental trials with groups of 32) latencies of departure (the time elapsed between the previous departure that led to $n_M = k$ and the next one that leads to $n_M = k + 1$). As these latencies are exponentially distributed, we hypothesize that a memoryless phenomenon is at stake. Thus that the probability per unit of time for one individual to depart is constant while the group configuration (the combination of $\mathrm{n_M}$ and $\mathrm{n_{S_S}}$) is the same. The individual experimental departure rate (μ) for a given value of $\mathrm{n_M}$ is estimated by computing the inverse of the mean experimental latency divided by $\mathrm{n_{S_S}}$. We apply the same method to estimate the individual stopping rate (σ) because the distribution of latencies for a given value of $\mathrm{n_{S_T}}$ also follows an exponential law. This estimation process is also detailed in [42].
