# Supplementary material for: Imitation Combined with a Characteristic Stimulus Duration Results in Robust Collective Decision-Making
Source: PLoS One. 2015 Oct 14;10(10):e0140188. doi: 10.1371/journal.pone.0140188 (PMC4605660; doi:10.1371/journal.pone.0140188)
Supplement: S4 Text — (PDF) [file pone.0140188.s004.pdf]

S4 Text

Details on the parameter estimation. Following Pillot *et al.* [42], we quantify the individual response function, i.e. the departure rate μ (respectively the stop rate σ) considering that sheep are stimulated to move (respectively to stop) by all moving (stopped) sheep but inhibited by all stopped (moving) ones with

$$\mu = \alpha \frac{nM^{\beta}}{nS_S^{\gamma}}, \tag{S4.1}$$

and

$$\sigma = \alpha' \frac{nS_T^{\beta'}}{nM^{\gamma'}}. \tag{S4.2}$$

Previously, Pillot *et al.* never recorded cases where no collective departure was observed. The parameter estimation we perform is of two types for departing and stopping phases because we want to get parameters values allowing to predict lack of collective departures (which is highly dependent on the first followers' latencies), but also to account for the dual combination of mimetic rules in order to get coherent description of the two phases when increasing the group size and the number of initiators. About the departing phase, the estimation of the parameters that best fit the experimental data is a non-trivial task. We want equation (3) to produce a value of commitment C close to the experimental data (Fig. 3D), while equation (1) to reproduce as faithfully as possible the temporal patterns of the departure and the stopping phases respectively (Fig. 3E). Let's consider first the commitment C. Equation (3) which models C depends only on α and γ for i = 1. We adjust α and γ to obtain the best fitting of equation (3), and then we have only one free parameter to play with (β) to tune equation (1). We kept the values of α and γ that minimize the error (as calculated by the average of the squares of differences) on C, and looked for a value of β minimizing the error on the departure rates. With respect to experimental departure rates, we modify the procedure followed by Pillot *et al.* in order to take into account both trials with and without collective departures. The modified procedure computes the survival analysis on the distribution of the departure latency of the first followers including those censored by the time spent by initiators to reach the target in trials where they were not followed (n = 6). Because the distribution of latencies with censorship follows an exponential function, we then are able to calculate the associated time constant and thus the estimated rate for first followers in groups of 32 (estimated rate without censoring: 0.19, with censoring: 0.08; time constant of the associated exponential without censoring: 5.22 s, with censoring: 11.56 s; S4 Fig). We use this corrected rate in figures and parameter estimation. This process of parameter estimation gives the following values: $\alpha = 90.1$, $\beta = 2.5$ and $\gamma = 3$. In the case of the stopping phase, we need to estimate parameters only for equation (2). We fit the equation (2) on the experimental stop rates, giving $\alpha' = 0.23$, $\beta' = 0.53$ and $\gamma' = 0.41$. The range of values obtained for β, γ, β', and γ' are larger than zero, which is in accordance with the hypotheses considering a promoting role of $\mathrm{n_M}$ ($\mathrm{n_{S_T}}$) and a

inhibiting role of n_{S_S} (n_M) for the transition from S_S to M and from M to S_T, respectively.
