# Supplementary material for: Imitation Combined with a Characteristic Stimulus Duration Results in Robust Collective Decision-Making
Source: PLoS One. 2015 Oct 14;10(10):e0140188. doi: 10.1371/journal.pone.0140188 (PMC4605660; doi:10.1371/journal.pone.0140188)
Supplement: S5 Text — (PDF) [file pone.0140188.s005.pdf]

S5 Text

Details on the calculation of predicted $\mathbf{n_M(t)}$. Given $\mu(t)$ and $\sigma(t)$, it is straightforward to obtain $n_M(t)$. Let us initially focus on $n_M(t)$ during the departure phase. Let t_1, the departure time of the initiator and t_2, t_3 ... $t_{n_M=N}$ the departure times of the first, second ... N-1th followers. Assume that at time $t_1 = 0$, the initiator departs from the group. The time t_2 - t_1 we have to wait to observe the departure of the first follower is exponentially distributed and characterized by an average time:

$$\frac{1}{\tilde{\mu}(n=1,N)} = \frac{1}{(N-1)^{1-\gamma}\alpha.1^{\beta}}. \tag{S5.1}$$

Now we want to know at which time we will observe a second follower (to reach $n_M = 3$). We know that t_3 - t_2 is exponentially distributed and that the average t_3 - t_2 is given by:

$$\frac{1}{\tilde{\mu}(n=2,N)} = \frac{1}{(N-2)^{1-\gamma}\alpha.2^{\beta}}. \tag{S5.2}$$

More generally, the average time between the n follow event and the n-1 follow event is given by:

$$t_{n+1} - t_n = \frac{1}{\tilde{\mu}(n,N)} = \frac{1}{(N-n)^{1-\gamma}\alpha.n^{\beta}} \tag{S5.3}$$

This means that the average time at which the n_M event occurs can be expressed as:

$$t_{nM} = \sum_{n=1}^{nM-1} t_{n+1} - t_n = \sum_{n=1}^{nM-1} \frac{1}{\tilde{\mu}(n,N)} = \sum_{n=1}^{nM-1} \frac{1}{(N-n)^{1-\gamma}\alpha.n^{\beta}} \tag{S5.4}$$

This allows us to build a list as shown in S1 Table.

The list provides all the information we are interested in. However, notice that we have not derived an analytical expression for $n_M(t)$, but an expression of the form:

$$t = \sum_{n=1}^{nM-1} \frac{1}{\tilde{\mu}(n,N)} = f(nM) \tag{S5.5}$$

An explicit expression for $n_M(t)$ requires finding the inverse of f, which we denote f^{-1}, in order to obtain $f^{-1}(t) = n_M$.

The derivation of the curve $n_M(t)$ in the stopping phase goes along similar lines. First, we assume that $t_1 = \tau$, with τ being the time required by the initiator to arrive at the target position. The time difference t_2 - t_1 refers to the time elapsed between the stop of the initiator and the first stop of a naive

individual. As before, this time difference is exponentially distributed, and its average is given by:

$$\frac{1}{\tilde{\sigma}(n=1,N)} = \frac{1}{(N-1)^{1-\gamma'}\alpha'.1^{\beta'}} \tag{S5.6}$$

For the time difference t_3 - t_2 between the first and second stop (of naive individuals), the average is given by:

$$\frac{1}{\tilde{\sigma}(n=2,N)} = \frac{1}{(N-2)^{1-\gamma'}\alpha'.2^{\beta'}} \tag{S5.7}$$

The generalization reads simply as:

$$t_{n-1} - t_n = \frac{1}{\tilde{\sigma}(n,N)} = \frac{1}{(N-n)^{1-\gamma'}\alpha'.n^{\beta'}} \tag{S5.8}$$

As before, we can obtain the time from the previous expression. Before doing so, and since we are interested in the temporal evolution of the number of individuals in state M, *i.e.* n_M, we introduce a change of variable. The first individual that stops is the initiator, at which point n_M = N - 1. With the first naive individual to stop, n_M = N - 2. In short, we can either refer to the first naive individual to stop, the second, etc., as to n_M = N - 2, n_M = N - 3, etc. The time difference can be relabelled as t_{N-2} - t_{N-1} for the first stop of a naive individual, t_{N-3} - t_{N-2} for the second stop of a naive individual, etc., and define $t_{N-1} = \tau$. We use this fact for n_M < N - 1 to express :

$$t_{nM} - \tau = \sum_{k=nM}^{N-2} t_k - t_{k+1} = \sum_{k=nM}^{N-2} \frac{1}{\tilde{\sigma}(k,N)} = \sum_{k=nM}^{N-2} \frac{1}{(k+1)^{1-\gamma'}\alpha'(N-(k+1))^{\beta'}} \tag{S5.9}$$

From this it is obvious that:

$$t_n M = \tau + \sum_{k=nM}^{N-2} \frac{1}{(k+1)^{1-\gamma'}\alpha'(N-(k+1))^{\beta'}} \tag{S5.10}$$

As before, we can make use of this expression to build a list as shown in S2 Table.

Notice that knowing the average duration of the departing phase given by:

$$t_N = \sum_{n=1}^{N-1} \frac{1}{(N-n)^{1-\gamma'}\alpha'.n^{\beta'}} \tag{S5.11}$$

the duration of the collective motion phase is simply $\tau - t_N$.
