# Supplementary material for: Imitation Combined with a Characteristic Stimulus Duration Results in Robust Collective Decision-Making
Source: PLoS One. 2015 Oct 14;10(10):e0140188. doi: 10.1371/journal.pone.0140188 (PMC4605660; doi:10.1371/journal.pone.0140188)
Supplement: S6 Text — (PDF) [file pone.0140188.s006.pdf]

S6 Text

Details on the calculation of the commitment (equation (3)). The commitment is defined as the probability of observing a naive abiding by the departure of the initiator. Here, we assume a general case where we have an arbitrary number of initiators i. This means that we have N - i naive individuals. The probability per time unit per naive individual to switch from S_S to M is given by μ. Since N - i individuals can potentially decide to follow the initiator(s), the probability per time unit (*e.g.*, seconds) to observe the first naive to depart is given by:

$$\tilde{\mu} = (N - i)\mu = \alpha . i^{\beta}(N - i)^{1-\gamma} \tag{S6.1}$$

From this expression we can obtain the probability that no naive will depart during time t and that it happens between t and t + dt. This probability takes the form: $e^{-\tilde{\mu}t}\tilde{\mu}.dt$. For a given τ (time required to arrive to the target), the probability that a following event occurs for t > τ is simply:

$$\int_{\tau}^{\infty} e^{-\tilde{\mu}t}\tilde{\mu}.dt = e^{-\tilde{\mu}\tau} \tag{S6.2}$$

The commitment, denoted as C, is the probability that a naive departed during τ, and thus it is expressed by:

$$C(\tau) = 1 - e^{-\tilde{\mu}\tau} \tag{S6.3}$$

Now, for simplicity, we assume that the distribution of time τ, denoted by $p(\tau)$ is an uniform distribution between the experimentally observed τ_{min} and τ_{max} (i.e., $p(\tau) = \frac{1}{\tau_{max} - \tau_{min}}$) and compute the average $C(\tau)$:

$$C = \int_{\tau_{min}}^{\tau_{max}} C(\tau)p(\tau).d\tau = 1 - \frac{1}{(\tau_{max} - \tau_{min})\tilde{\mu}}(e^{-\tilde{\mu}\tau_{min}} - e^{-\tilde{\mu}\tau_{max}}) \tag{S6.4}$$

This is the expression we use to calculate the expected commitment that we compare with the experimental observation. Exploring the model allows to check the effect of adding initiators on the value of commitment as a function of group size (S5 FigA). Also, for a given group size (here N = 100), we see that the commitment value increases non-linearly with the number of initiators (S5 FigB). Finally, we were able to compute the number of initiators i needed to recruit all group members (S5 FigC).
