# Supplementary material for: Imitation Combined with a Characteristic Stimulus Duration Results in Robust Collective Decision-Making
Source: PLoS One. 2015 Oct 14;10(10):e0140188. doi: 10.1371/journal.pone.0140188 (PMC4605660; doi:10.1371/journal.pone.0140188)
Supplement: S1 Table — (PDF) [file pone.0140188.s007.pdf]

n_M	Time
1	t_1
2	t_2
...	...
...	...
N	t_N

S1 Table. List of times obtained by Equation S5.4.
