# Supplementary material for: Imitation Combined with a Characteristic Stimulus Duration Results in Robust Collective Decision-Making
Source: PLoS One. 2015 Oct 14;10(10):e0140188. doi: 10.1371/journal.pone.0140188 (PMC4605660; doi:10.1371/journal.pone.0140188)
Supplement: S2 Table — (PDF) [file pone.0140188.s008.pdf]

| $n_M$ | Time |
|---|---|
| $N-1$ | $t_{N-1}$ |
| $N-2$ | $t_{N-2}$ |
| ... | ... |
| ... | ... |
| 0 | $t_0$ |

**S2 Table.** List of times obtained by Equation S5.10.
